# Inappropriate prescribing and association with readmission or mortality in hospitalised older adults with frailty: a systematic review and meta-analysis

**DOI:** 10.1186/s12877-024-05297-3

**Published:** 2024-08-29

**Authors:** Joshua M. Inglis, Gillian Caughey, Tilenka Thynne, Kate Brotherton, Danny Liew, Arduino A. Mangoni, Sepehr Shakib

**Affiliations:** 1 Department of Clinical Pharmacology, Flinders Medical Centre and Flinders University, Adelaide, SA Australia; 2 Adelaide Medical School, University of Adelaide, Adelaide, SA Australia; 3 Registry of Senior Australians, South Australian Health and Medical Research Institute, Adelaide, SA Australia; 4 Department of General Medicine, Royal Adelaide Hospital, Adelaide, SA Australia; 5 Department of Clinical Pharmacology, Royal Adelaide Hospital, Adelaide, SA Australia

**Keywords:** Aged, Inappropriate prescribing, Middle aged, Mortality, Hospital Readmission, Frailty

## Abstract

**Background:**

Inappropriate prescribing (IP) is common in hospitalised older adults with frailty. However, it is not known whether the presence of frailty confers an increased risk of mortality and readmissions from IP nor whether rectifying IP reduces this risk. This review was conducted to determine whether IP increases the risk of adverse outcomes in hospitalised middle-aged and older adults with frailty.

**Methods:**

A systematic review was conducted on IP in hospitalised middle-aged (45–64 years) and older adults (≥ 65 years) with frailty. This review considered multiple types of IP including potentially inappropriate medicines, prescribing omissions and drug interactions. Both observational and interventional studies were included. The outcomes were mortality and hospital readmissions. The databases searched included MEDLINE, CINAHL, EMBASE, World of Science, SCOPUS and the Cochrane Library. The search was updated to 12 July 2024. Meta-analysis was performed to pool risk estimates using the random effects model.

**Results:**

A total of 569 studies were identified and seven met the inclusion criteria, all focused on the older population. One of the five observational studies found an association between IP and emergency department visits and readmissions at specific time points. Three of the observational studies were amenable to meta-analysis which showed no significant association between IP and hospital readmissions (OR 1.08, 95% CI 0.90–1.31). Meta-analysis of the subgroup assessing Beers criteria medicines demonstrated that there was a 27% increase in the risk of hospital readmissions (OR 1.27, 95% CI 1.03–1.57) with this type of IP. In meta-analysis of the two interventional studies, there was a 37% reduced risk of mortality (OR 0.63, 95% CI 0.40-1.00) with interventions that reduced IP compared to usual care but no difference in hospital readmissions (OR 0.83, 95% CI 0.19–3.67).

**Conclusions:**

Interventions to reduce IP were associated with reduced risk of mortality, but not readmissions, compared to usual care in older adults with frailty. The use of Beers criteria medicines was associated with hospital readmissions in this group. However, there was limited evidence of an association between IP more broadly and mortality or hospital readmissions. Further high-quality studies are needed to confirm these findings.

**Supplementary Information:**

The online version contains supplementary material available at 10.1186/s12877-024-05297-3.

## Introduction

Inappropriate prescribing (IP) is common, occurring in 47–56% of hospitalised older adults [1]. The prevalence of IP in hospitalised middle-aged adults has not been well studied but is reported to occur in one-fifth of community-dwelling middle-aged adults [2, 3]. Individuals who have been hospitalised are likely to have a higher risk of poor health outcomes compared to those in the community [4]. Hospitalisation also represents an opportunity for medication reconciliation and deprescribing which may improve outcomes. The definition of IP is broad and includes potentially inappropriate medicines (PIMs), potential prescribing omissions (PPOs) and unsuitable combinations of medicines [5]. PPOs are defined as the omissions of medicines that may have benefit for a patient [6]. There are numerous tools to identify PIMs and PPOs, which include the Screening Tool of Older Persons’ Prescriptions (STOPP), the Screening Tool to Alert to Right Treatment (START) and the Beers criteria [5, 7]. Multimorbidity, defined as the presence of two or more long term health conditions, is also common, occurring in 16–98% of adults [8–10]. Unsuitable combinations of medicines may result from the application of multiple disease-specific guidelines in patients with multimorbidity [11–14]. This may lead to IP through drug-drug or drug-disease interactions in such patients.

Frailty is a health state characterised by increased vulnerability to external stressors and generally affects middle-aged to older adults [15, 16]. There are multiple validated definitions of frailty for research and clinical purposes that have been associated with adverse outcomes [15]. Frailty is a related but distinct concept from multimorbidity although the two are commonly concurrent [17]. Frailty is common in hospitalised older adults with a pooled prevalence of 47% [18]. There is some evidence that frailty may occur in middle-aged adults with several frailty parameters being present in more than 20% of middle-aged adults attending an outpatient clinic in one study [16]. Older adults and those with frailty are likely to have reduced physiological reserve and altered pharmacokinetics and pharmacodynamics [19, 20]. This augments the risk of adverse outcomes from IP [13, 21].

A previous systematic review on potentially IP in hospitalised older adults did not show an association with all-cause mortality nor hospital readmissions. The included studies with an average follow-up of 14 months [2]. However, this review used a narrow definition of IP that did not include drug-drug or drug-disease interactions which are known to be common in adults with multimorbidity [22]. It also did not include middle-aged adults, the largest group of patients with multimorbidity in absolute terms who may also be frail [16, 23]. Furthermore, there was no subgroup analysis for patients with frailty who may be at increased risk of adverse outcomes [23].

The aim of the systematic review was to determine whether IP is associated with an increased risk of readmission and mortality in middle-aged and older adults with frailty and whether interventions addressing IP change that risk.

## Methods

The systematic review was conducted and reported in accordance with the Preferred Reporting Items for Systematic Reviews and Meta-Analyses (PRISMA) statement [24]. The protocol was prospectively registered on PROSPERO (CRD42022341998).

### Inclusion criteria

#### Participants

The review considered studies involving hospitalised individuals who were middle-aged (45–64 years) and older (≥ 65 years) where it was explicitly stated that participants were frail. The age ranges for middle-aged and older adults was in accordance with accepted definitions [25]. Studies were required to have at least 80% of participants meeting the criteria for middle or older aged adults with frailty where the outcomes of mortality or readmissions from these subgroups could be extracted. This threshold was chosen to ensure that the majority of participants fell within the target range so that our findings would be generalisable to the intended population. The use of a threshold is consistent with guidance in the Cochrane Handbook and was possible since participant age is consistently reported in these studies [26]. This same threshold has been used in previously published systematic review protocols in the area of inappropriate prescribing [27]. 

#### Exposure of interest

Studies reporting IP included any one of three concepts: (i) inappropriate medicines; (ii) prescribing omissions; and (iii) drug interactions including drug-drug interactions, drug-disease interactions, duplication of medicines and treatment-related medicine conflicts [2, 28, 29]. These three concepts were selected based on the conceptualisation of IP in a previously published systematic review to ensure all types of IP were captured in the review [30]. Studies evaluating a single inappropriate medicine, medicine omission or drug-interactions were also included. Studies assessing incorrect doses, incorrect frequencies, or incorrect durations of prescribing or duplication of medications were also included. In instances of uncertainty, the Australian Medicines Handbook was consulted, which is the primary source of medicine information for prescribers In Australia [31]. Prescribing inconsistent with the recommendations in this resource was considered inappropriate. This method of adjudication is consistent with a previously published systematic review in this area [22].

#### Outcomes

The outcomes of interest included mortality (using any measure including all-cause mortality, non-cancer mortality and cause-specific mortality) and hospital readmission (including presentations to the emergency department, all-cause readmission, cause-specific readmission, and readmission for medicine-related problems). Readmissions were chosen as the outcome since this review only considered studies involving hospitalised individuals. There were no other outcomes assessed. There was no minimum duration of follow up.

#### Types of studies

Included studies must have a comparator group. The review included observational studies as well as experimental and quasi-experimental study designs such as randomised controlled trials (RCTs), non-randomised trials, before-after studies and interrupted time-series studies which reported on the risk associated with an intervention. Review articles, case reports and case series were excluded.

#### Search strategy

A three-step search strategy was utilised to locate both published and unpublished studies. First, an initial limited search of MEDLINE (PubMed) was undertaken to identify articles on the topic. The text words contained in the titles and abstracts of relevant articles, and the index terms used to describe the articles were used to develop a full search strategy for the databases to be searched. These comprised MEDLINE (OVID), CINAHL (EBSCO), EMBASE (OVID), World of Science, SCOPUS, and the Cochrane Library. These databases were initially searched from inception to 12 July 2022. The review was updated to 12 July 2024 with an additional search of MEDLINE (OVID). The ‘grey literature’ was searched using Google Scholar. Only studies published in English were included.

The search terms were developed in consultation with a librarian specialising in health databases (Appendix A). The search strategy was adapted from Mekonnen et al. and Sirois et al. [2, 32]. Published criteria for inappropriate medicines and prescribing omissions were included in the search. These were identified from a systematic review on the topic, criteria identified from the initial search of MEDLINE (PubMed) and the authors’ knowledge of published criteria for use in this population [2].

#### Study selection

All identified citations were collated and uploaded into Covidence (Covidence, Melbourne, Australia) and duplicates removed. Following a pilot test, titles and abstracts were screened by two independent reviewers (JI and TT or KB) for assessment against the inclusion criteria. Potentially relevant studies were retrieved in full.

The full text of selected citations was assessed in detail against the inclusion criteria by two independent reviewers (JI and KB). Reasons for exclusion of papers were recorded. Any disagreements that arose between the reviewers at each stage of the selection process were resolved through discussion with a third investigator (AM).

#### Assessment of methodological quality

The methodological quality was assessed using the Joanna Briggs Institute (JBI) Critical Appraisal Checklists for RCTs, cohort studies, analytical cross-sectional studies and case-controlled studies [33]. This was undertaken by two independent reviewers (JI and KB). Any uncertainty was resolved through discussion with a third investigator (AM). Authors were contacted to request missing or additional data for clarification, as required. Studies with scores < 50% were considered to have high risk of bias, 50–70% were considered to have moderate risk of bias and > 70% were considered to have low risk of bias. All studies, regardless of the results of their methodological quality, underwent data extraction and synthesis.

#### Data extraction

A predefined data extraction template was developed and included details about the study design, sample size, age of participants, inclusion criteria, study duration, follow up period, intervention where appropriate, definition used to determine prescribing appropriateness, adverse outcomes, and measure of frailty (Appendix B). Authors were contacted to request missing or additional data as required. The data extraction template was initially validated by two independent reviewers and then data extraction completed by one author (JI). Once the primary data extraction was complete, all authors reviewed the data extracted for each of the studies.

#### Data synthesis

Studies were pooled in statistical meta-analysis using Stata 17 (StataCorp LLC, College Station, TX, USA). Effect sizes expressed as odds ratios with 95% confidence intervals (95% CIs) were calculated. Where effect estimates and standard errors were not available, they were calculated from crude data along with 95% CIs. Where a study reported multiple odds ratios based on time periods, the longest follow-up time was used to capture long term effects [34]. 

In a random effects model, untransformed effect-size estimates were used to calculate the weighted mean correlation coefficient. The random effects model was selected given the anticipated clinical heterogeneity of the studies. Heterogeneity was assessed statistically using the Cochrane Q test and I². Where meta-analysis was not possible, the findings were presented in narrative form.

## Results

**Fig. 1:**
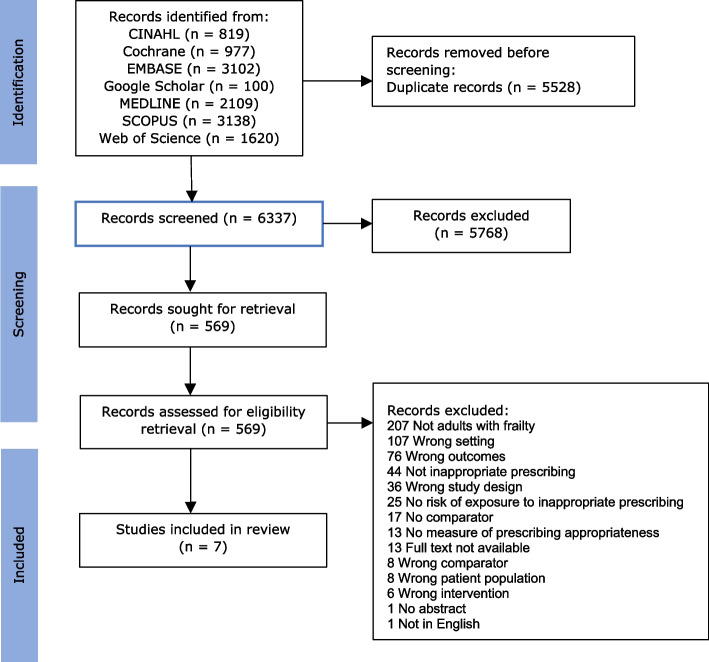
PRISMA 2020 flow diagram

A total of 11,865 records were identified. After removing duplicates, 6,337 records were screened by title and abstract. Of these, 569 required full-text review, 576 were excluded with the most common reasons being that they were not in adults with frailty (*n* = 207), they were in the wrong setting (*n* = 107), had the wrong outcomes (*n* = 76) or were not assessing IP (*n* = 44). A total of seven studies met the inclusion criteria and were included in this review (Fig. 1). Five studies were observational [21, 35–38] and two interventional [39, 40]. All included studies had older individuals with frailty as the population or a subgroup. The sample of participants with frailty in the five observational studies ranged from 200 to 1762 and in the interventional studies ranged from 130 to 240.

### Risk of bias

Of the five observational studies, four were at low risk of bias and one was at moderate risk of bias. The two interventional studies were at moderate and low risk of bias (Tables 1, 2 and 3).

**Table 1:** Critical appraisal of randomized controlled trials

Citation	True Randomisation	Allocation Concealment	Treatment Groups Similar at Baseline	Participants Blind to Treatment Assignment	Those Delivering Treatment Blind to Treatment Assignment	Outcome Assessors Blind to Treatment Assignment	Treatment Groups Treated Identically other than Intervention of Interest	Follow up Complete or Differences Described	Participants Analysed in Groups to which they were Randomised	Outcomes Measures in Same Way for Both Groups	Outcomes Measures in Reliable Way	Appropriate Statistical Analysis	Trial Design Appropriate	Total Score	Quality Assessment
Curtin et al. 2020 [40]	Yes	No	Yes	No	No	Yes	Yes	No	Yes	Yes	Yes	Yes	Yes	9/13	Moderate
Schapira et al. 2022 [39]	Yes	Yes	Yes	No	No	Yes	Yes	Yes	Yes	Yes	Yes	No	Yes	10/13	Low

**Table 2:** Critical appraisal of case control studies

Citation	Groups Comparable Other Than Presence or Absence of Disease	Cases and Controls Appropriately Matched	Same Criteria to Identify Cases and Controls	Standard, Valid and Reliable Exposure Measurement	Exposure Measurement Same for Cases and Controls	Confounding Factors Identified	Strategies to Deal with Confounding Factors	Outcomes Assessed in Standard, Valid and Reliable Way	Exposure Period Long Enough to be Meaningful	Appropriate Statistical Analysis	Total Score	Risk of Bias
Cheong et al. 2020 [37]	Unclear	No	No	Yes	Yes	Yes	Yes	Yes	Yes	Yes	7/10	Moderate

**Table 3:** Critical appraisal of cohort studies

Citation	Groups Similar and Recruited from Same Population	Exposures Measured Similarly for Exposed and Unexposed Groups	Exposure Measures in Valid, Reliable Way	Confounding Factors Identified	Strategies to Deal with Confounding Factors	Participants Free of Outcome at Start of Study	Outcomes Measured in Valid, Reliable Way	Follow up Time Reported and Sufficiently Long Enough	Follow up Complete	Strategies to Address Incomplete Follow Up	Appropriate Statistical Analysis	Total Score	Risk of Bias
Bennett et al. 2014 [21]	Yes	Yes	Yes	Yes	Yes	Yes	Yes	Yes	Yes	N/A	Yes	10/10	Low
de Almeida et al. 2020 [36]	Yes	Yes	Yes	Yes	Yes	Yes	Yes	Yes	Yes	N/A	Yes	10/10	Low
Forget et al. 2020 [35]	No	Yes	Yes	Yes	Yes	Yes	Yes	Yes	No	Unsure	Yes	8/11	Low
Liang et al. 2023 [38]	Unclear	Yes	Yes	Yes	Yes	Yes	Yes	Yes	Yes	No	Yes	9/11	Low

In two of the five observational studies, the baseline characteristics of the groups were different between those exposed and not exposed to IP [35, 36] (Table 3). In Liang et al., it was unclear where the baseline characteristics of the frailty subgroup exposed and not exposed to IP were similar [38]. In Cheong et al., comparability between groups was unable to be determined, other than the presence or absence of IP, and cases and controls were not appropriately matched (Table 2) [37]. In Forget et al. it was unclear whether follow up was complete, how many participants were lost to follow up and what strategies were in place to address this [35]. The authors were contacted to clarify this, but no response was received.

Although all five observational studies adjusted for covariates, the individual covariates used varied between studies. Most studies adjusted for age, sex and comorbidities either using number of comorbidities or the Charlson comorbidity score. Three studies adjusted for functional status (using either activities of daily living [ADLs], instrumental ADLs or the Katz score), two studies adjusted for living situation and two studies adjusted for admitting unit. One study adjusted for medication-related factors (e.g. number of as needed drugs, number of high-risk drugs, diuretics prescribed, and hyperpolypharmacy) and one adjusted for renal function.

Of the interventional studies, Curtin et al. did not blind assessors to the treatment assignment, follow up was incomplete and the characteristics of those lost to follow up between groups was not described (Table 1) [40].

### Characteristics of included studies

The details of the studies are outlined in Table 4. The studies identified were RCTs (*n* = 2), retrospective cohort studies (*n* = 3), a prospective cohort study (*n* = 1) and a case-control study (*n* = 1). Studies were conducted in Argentina, Australia, Canada, Ireland, Portugal, Taiwan, and the UK.

**Table 4:** Details of included studies

Study	Study Design	Risk of Bias	Sample Size	Age (mean and SD)	Population	Study Duration and Follow Up Period	Intervention	Measure of Prescribing Appropriateness	Adverse Outcomes	Measure of Frailty	Outcomes
Schapira et al. 2022 [39]	RCT	Low	240	Usual care 85.4 ± 6.7 Intervention 85.9 ± 6.6	Adults ≥ 75 years and older who had an unplanned hospital admission to the internal medicine service and fulfilled the definition of frailty according to the Frailty Index	August 2015 to December 2016, follow up 6 from discharge	In-hospital geriatric co-management combined with interdisciplinary transitional care intervention	Nil	Significant reduction in 30-day hospital readmissions and 6-month ED visits but no difference in 6-month mortality	Frailty Index	Hospital readmissions at 30 days 42 patients (35%) with usual care compared to 22 patients (18.3%) with the intervention *P* = 0.004* ED visits 72 patients (60.0%) with usual care compared to 52 patients (43.5%) with the intervention *P* = 0.010* Mortality 42 patients (35.0%) with usual care compared to 31 (25.8%) with the intervention *P* = 0.123
Curtin et al. 2020 [40]	RCT	Moderate	130	85.1 ± 5.7	Hospitalised adults ≥ 75 years with acute medical or surgical illness with advanced frailty and polypharmacy (5 or more medicines)	March 2018 to April 2019, follow up 3 months	STOPPFrail-guided deprescribing advice	STOPPFrail-defined PIMs	No significant difference in 3-month ED visits, unplanned hospital admissions or deaths	Clinical Frailty Scale	ED presentations RR 0.60 (0.15–2.41) *P* = 0.72 Unplanned hospital admissions RR 1.80 (0.64–5.08) *P* = 0.27 Deaths RR 0.67 (0.35–1.27) *P* = 0.22
Bennett et al. 2014 [21]	Prospective cohort	Low	204 (103 with frailty)	80.5 ± 8.3	Adults ≥ 60 years of age admitted with a fall to a tertiary referral teaching hospital	June 2012 to March 2013, follow up 2-months from discharge	-	Fall-risk-increasing drugs and drug–drug interactions	No significant difference in 2-month hospital readmissions	Edmonton Frail Score	Hospital readmissions Adjusted OR 1.0 (0.8–1.4) for fall-risk-increasing drugs *P* > 0.05 but no value given Adjusted OR 0.9 (0.3–2.5) for drug-drug interactions *P* > 0.05 but no value given
Forget et al. 2020 [35]	Retrospective cohort	Low	300	Median 72 IQR 69–76	Adults ≥ 65 years at the time of preoperative evaluation, awaiting a major elective surgery with an expected hospitalization stay of > 24 h and undergoing an evaluation in the preoperative clinic	January 2017 to January 2018, follow up 90 days from discharge	-	MedSafer tool including Beers Criteria, STOPP, Choosing Wisely Recommendations and emerging evidence	No significant difference in 90-day ED visits	Clinical Frailty Scale	ED visits Adjusted OR 1.51 (0.90–2.52) *P* = 0.119
de Almeida et al. 2020 [36]	Retrospective cohort	Low	327	82 ± 8	Patients admitted to an internal medicine ward with a previous diagnosis of atrial fibrillation and discharged with a direct oral anticoagulant	January 2016 to December 2016, follow up to 1 year from discharge	-	Inappropriate dosing of direct oral anticoagulants	No significant difference in 1-year mortality	None	Mortality Adjusted HR 1.4 (0.9–2.1) *P* = 0.110
Liang et al. 2022	Retrospective cohort	Low	3061 (1762 with frailty)	77.2 ± 8.4	Patients aged ≥ 65 years who were admitted to the internal medicine ward of a tertiary teaching hospital in southern Taiwan	April and December of 2017, followed for up to 6 months	-	2015 Beers Criteria	Significant increase in readmissions within 1 and 3 months but not 6 months Significant increase in ED revisits within 1, 3 and 6 months	Multimorbidity frailty index	Readmissions within 1 month PIMs adjusted OR 1.41 (1.07–1.87) Readmission within 3 months PIMs adjusted OR 1.46 (1.16–1.83) Readmissions within 6 months PIMs adjusted OR 1.23 (0.99–1.54) ED revisits within 1 month PIMs adjusted OR 1.48 (1.13–1.93) ED revisits within 3 months PIMs adjusted OR 1.40 (1.12–1.76)) ED revisits within 6 months PIMs adjusted OR 1.26 (1.01–1.57)
Cheong et al. 2020 [37]	Case-control	Moderate	200	83.8 ± 5.68	Adults ≥ 75 years with unplanned medical admissions into a large teaching hospital	January 2015 to December 2015	-	High-risk medicines and Beers Criteria	No significant difference in repeated hospital admissions within study period of 1 year	None	Repeated readmission For PIMs – Adjusted OR 1.80 (0.88–3.68) For high risk drugs – Adjusted OR 0.87 (0.63–1.20)

Participants were mainly female in most studies (ranging from 35.2 to 73%), with the mean or median age from 72 to 85 years. Most of the studies were limited to older adults using various cut-offs including greater than 60, 65 or 75 years. Only one study included adults of all ages, however, the median age and distribution suggests that few if any middle-aged adults were included [36].

Studies differed with respect to the cohort of hospitalised patients included. These varied from all admitted patients to those with unplanned medical admissions, those admitted after a fall, those admitted with an acute medical or surgical illness, those with polypharmacy and those with atrial fibrillation on direct oral anticoagulants.

Although all studies were reported to be in a frail population, only five used validated measures of frailty. These were the Clinical Frailty Score (*n* = 2), the Frailty Index of Cumulative Deficits, (*n* = 1), the reported Edmonton Frail Score (*n* = 1) and the Multimorbidity Frailty Index (*n* = 1). One study used the modified Katz score, although this is a measure of functional status rather than frailty. Another used repeated readmissions given this was a feature of the Edmonton Frailty Scale, although the threshold used would not have equated to a classification of frailty in isolation using this scale [41, 42].

Of the interventional studies, one assessed in-hospital geriatric co-management combined with an interdisciplinary transitional care intervention, which included deprescribing advice using the Beers criteria compared to usual care [39]. The second study assessed the provision of deprescribing advice using the Screening Tool of Older Persons Prescriptions in Frail adults with limited life expectancy (STOPPFrail) criteria compared to usual care [40].

There were various tools used to identify inappropriate prescribing in the observational studies. Bennett et al. assessed the number of falls-risk increasing drugs and drug-drug interactions [21]. Forget et al. assessed the Beers criteria medicine, STOPP criteria, Choosing Wisely recommendations and emerging evidence [35]. de Almeida et al. assessed the inappropriate dosing of direct oral anticoagulants [36]. Cheong et al. assessed high-risk medicines and the Beers criteria [37]. Liang et al. assessed the Beers criteria [38]. 

Six studies assessed the outcome of hospital readmission or repeat Emergency Department (ED) visits and three assessed mortality. Most of the studies recruited patients over 1 year although follow up for these outcomes differed between studies. Shapira et al. assessed 30-day hospital readmissions, 6-month ED visits and 6-month mortality measured from time of discharge [39]. Curtin et al. assessed ED visits, unplanned readmissions and mortality within 3 months although it is unclear whether this was measured from the time of randomisation, the intervention or discharge [40]. The authors were contacted to clarify this, but no response was received. Bennett et al. assessed hospital readmissions at 2 months from time of discharge [21]. Forget et al. assessed ED visits, readmissions or mortality at 90 days from discharge [35]. de Almeida et al. assessed mortality within 1 year after discharge [36]. Cheong et al. assessed repeated hospital admissions within the study timeframe which was 1 year [37]. Liang et al. assessed readmissions and ED revisits for up to 6 months after discharge [38]. 

### Association of IP with readmissions and mortality

One of the five observational studies found an association between IP and ED visits and readmissions at specific time points. This was a retrospective cohort study of hospitalised adults ≥ 65 years in a teaching hospital in Taiwan [38]. It found an association between Beers criteria medicines and readmissions at 1 month (adjusted OR 1.41, 95% CI 1.07–1.87) and 3-months (adjusted OR 1.46, 95% CI 1.16–1.83) but not at 6-months (adjusted OR 1.23, 95% CI 0.99–1.54) in older adults with frailty. Beers criteria medicines were also associated with ED revisits at 1 month (adjusted OR 1.48 95% CI, 1.13–1.93), 3 months (adjusted OR 1.40, 95% CI 1.12–1.76) and 6 months (adjusted OR 1.26 95%, CI 1.01–1.57).

None of the remaining four observational studies found an association between IP and ED visits, readmissions, or mortality. A prospective cohort study of hospitalised individuals ≥ 60 years that included a subgroup with frailty identified using the reported Edmonton Frail Scale reported no difference in risk of hospitalisation for patients with frailty exposed to falls-risk-increasing drugs (FRID) (adjusted OR 1.0 95% CI 0.8–1.4) or drug-drug interactions (DDIs) (adjusted OR 0.9, 95% CI 0.3–2.5) [21]. A retrospective cohort study on older individuals aged ≥ 65 years with frailty undergoing a preoperative evaluation before major surgery reported that use of PIMs (identified using the MedSafer tool) was associated with longer length of stay but no difference in 90-day ED visits (adjusted OR 1.51, 95% CI 0.90–2.52) [35]. Assessment of inappropriate underdosing of direct oral anticoagulants in frail adults with atrial fibrillation who were admitted under internal medicine, reported no difference in mortality between those inappropriately underdosed and appropriately dosed (adjusted HR 1.4, 95% CI 0.9–2.1) [36]. A case-control study of older individuals aged ≥ 75 years with unplanned admissions, found no association between repeated readmissions and PIMs (adjusted OR 1.80, 95% CI 0.88–3.68) or high-risk drugs (adjusted OR 0.87, 95% CI 0.63–1.20) [37].

Of the two intervention studies, only one reported an intervention to reduce IP that lead to a reduction in hospital readmissions (OR 0.42, 95% CI 0.23–0.76) and ED visits (OR 0.51, 95% CI 0.31–0.85) in the 6 months after hospitalisation, but not mortality (OR 0.65, 95% CI 0.37–1.13) [39, 40]. However, in this study there were no significant differences in other adverse outcomes including pressure injuries, delirium, falls, physical restraint or chronic urinary catheterisation or chronic enteral feeding between the intervention and control groups [39]. In the second intervention study there was no difference in ED visits (RR 0.60, 95% CI 0.15–2.41, *P* = 0.72), unplanned readmission (RR 1.80, 95% CI 0.64–5.08, *P* = 0.27) or deaths (RR 0.67, 95% CI 0.35–1.27, *P* = 0.22) between deprescribing using STOPPFrail criteria and usual care [40].

### Meta-analysis

**Fig. 2:**
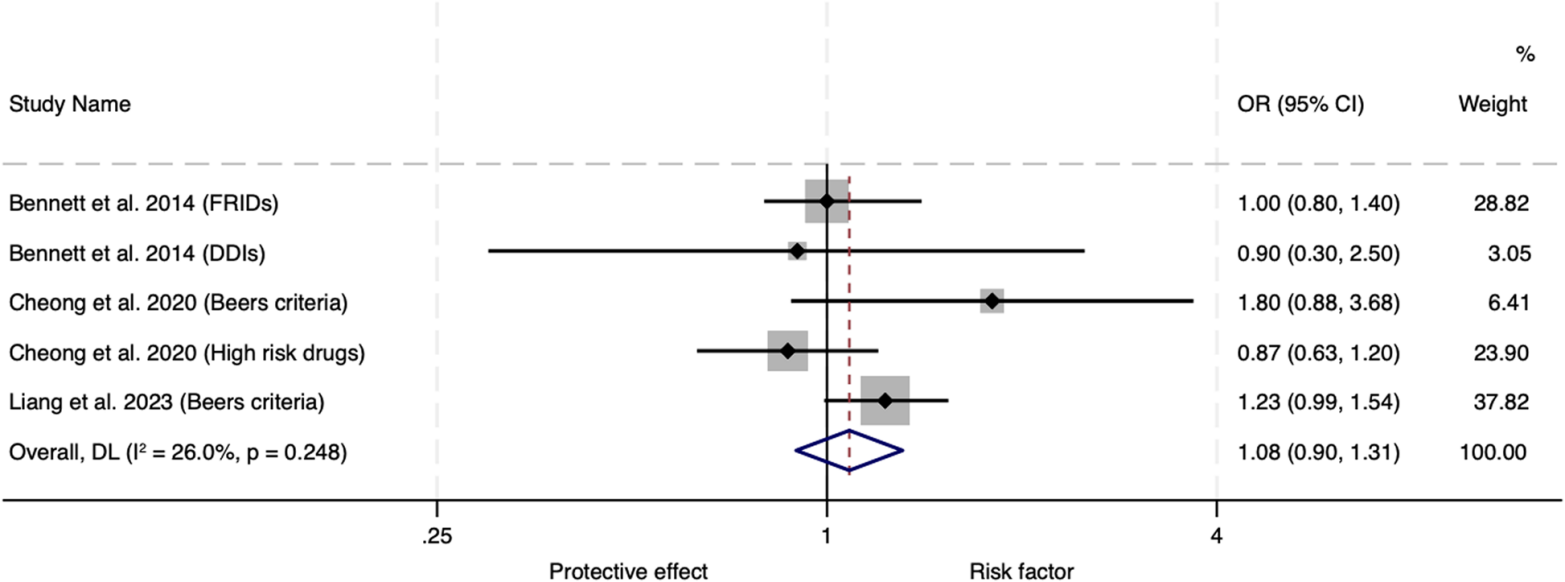
Meta-analysis of observational trials: adjusted odds ratios for hospital readmissions

**Fig. 3:**
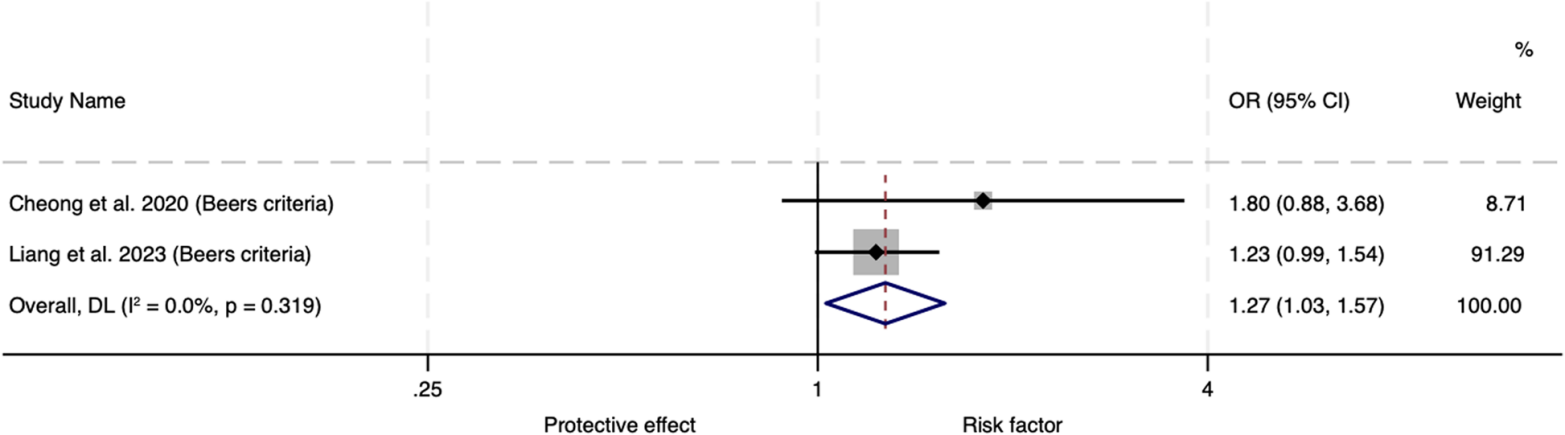
Meta-analysis of observational trials using Beers criteria as the definition of inappropriate prescribing: adjusted odds ratios for hospital readmissions

Three of the five observational studies were suitable for inclusion in a meta-analysis for the outcome of readmissions (Fig. 2). The remaining two observational studies did not report on the outcome of readmissions. There was no significant association between IP and hospital readmissions (OR 1.08, 95% CI 0.90–1.31) with minimal heterogeneity (I^2^ = 26.0%, *P* = 0.248). Sensitivity analyses were performed to ensure that the inclusions of two measures of IP from two study populations had not affected the results of the meta-analysis. Exclusion of any of the studies did not significant change the overall effect nor heterogeneity. A subgroup analysis was performed for the two of the observational studies using Beers criteria medicines. The odds ratio was 1.27 (95% CI 1.03–1.57) for readmissions in those receiving Beers criteria medicines compared to those not receiving these medicines (Fig. 3).

**Fig. 4:**
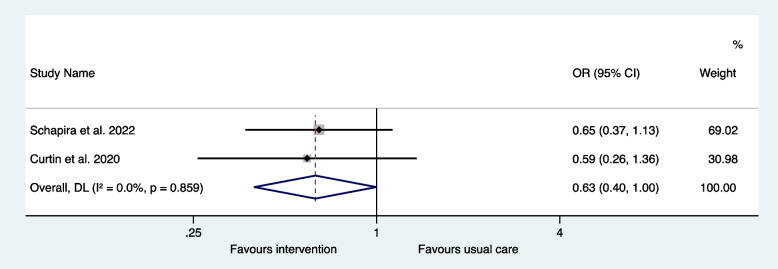
Meta-analysis of interventional trials: odds ratios for mortality

**Fig. 5:**
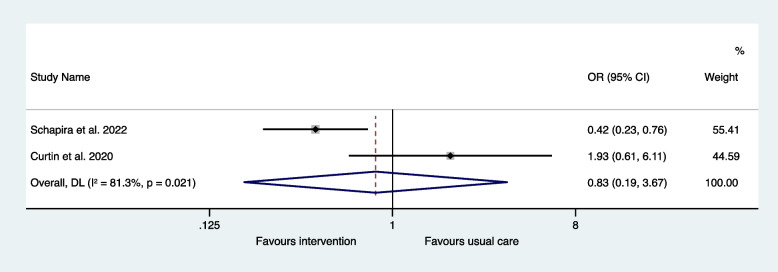
Meta-analysis of interventional trials: odds ratios for hospital readmissions

**Fig. 6:**
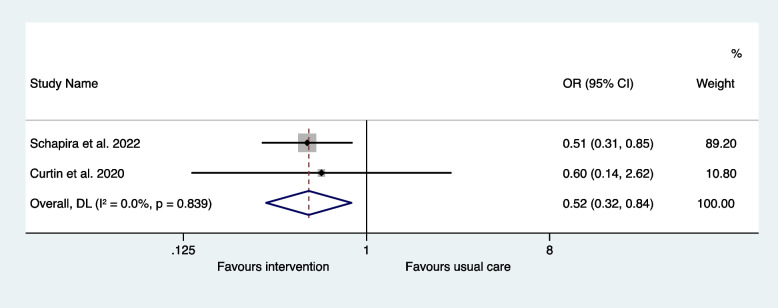
Meta-analysis of interventional trials: odds ratios for the subgroup of ED revisits

The two interventional studies were suitable for inclusion in a meta-analysis. The odds ratio for mortality was 0.63 (95% CI 0.40-1.00) in the interventions to reduce IP compared to usual care and there was minimal heterogeneity (I^2^ = 0.0%, *P* = 0.859) (Fig. 4). The odds ratio for hospital readmissions was 0.83 (95% CI 0.19–3.67) in the interventions to reduce IP compared to usual care and there was significant heterogeneity (I^2^ = 81.3%, *P* = 0.021) (Fig. 5). The odds ratio for the subgroup with ED revisits was 0.52 (95% CI 0.32–0.84) in the interventions to reduce IP compared to usual care and there was minimal heterogeneity (I^2^ = 0.0%, *P* = 0.839) (Fig. 6).

## Discussion

Interventions to reduce IP were associated with lower mortality compared with usual care in older individuals with frailty. Exposure to Beers criteria medicines was also associated with hospital readmissions in adults with frailty. However, there was minimal evidence to suggest an association between IP more generally and readmission or morality in the older population. No studies were identified in middle-aged individuals with frailty. Only five of the seven studies used validated tools to identify adults with frailty. This has the potential to influence results and future high-quality studies are needed using validated definitions of frailty with comprehensive adjustment for covariates including in middle-aged adults.

To the best of our knowledge, this is the first review to demonstrate that exposure to Beers medicines is associated with all-cause readmissions in older adults with frailty. A previous systematic review in hospitalised older adults had demonstrated an association with adverse drug events and adverse drug reactions as well as medication-related hospitalisation [2]. However, this study was unable to demonstrate an association between potentially inappropriate medicines across multiple tools and all-cause hospital readmissions. Another recent systematic review concluded that it was unclear whether potentially inappropriate medicine lists were associated with 30-day readmissions [43]. Only two of the six included studies demonstrated this association using a combination of the START/STOPP and the Beers criteria. This review only assessed readmissions at 30-days as opposed to our review where studies assessed this outcome up to 1 year after discharge. It is plausible that this association between Beers medicines and readmissions exists in older adults with frailty who may be at increased risk of medication-related harm due to altered pharmacokinetics and pharmacodynamics [19, 20, 44, 45].

There are various tools for the identification of adults with frailty in the clinical and research settings [15]. All of the studies included in this review that used validated methods of assessing frailty were using tools that have been associated with health outcomes [15, 46]. However, there were two studies that used unvalidated methods of identifying patients with frailty which may reflect the difficulties in applying these tools to real-world datasets. Hospitalised adults with frailty may reflect a higher-risk group more likely to benefit from deprescribing [4]. Systematic reviews of broader populations have seldom shown improvements in mortality with interventions that reduce IP [47–50]. The assessment of frailty would ideally be incorporated into routine clinical care to assist with the identification of such patients and aid future research [51]. The identification of frailty using ICD-10 codes in routinely collected hospital coding data may be applied to large datasets to facilitate research in this area [52, 53].

The heterogeneity of interventions to reduce IP and lack of statistical power between the interventional studies may account for the differing outcomes. Only one of the two interventional studies showed reductions in readmission and ED visits. The intervention in this study was geriatric co-management combined with an interdisciplinary transitional care intervention which included deprescribing of medicines from the Beers criteria [39]. The remaining study assessed deprescribing using the STOPPFrail criteria [40]. Despite 90% percent of patients having a deprescribing recommendation and 88% being implemented, there were no difference in the outcomes of readmissions, ED visits or mortality between groups. However, the trial was insufficiently powered for these outcomes. Although there was a significant reduction in ED visits and mortality with the interventions in meta-analysis, it is unclear whether this result was attributable to deprescribing or other aspects of the interventions. This is particularly the case for the study that included deprescribing as part of a comprehensive geriatric assessment which is known to reduce the risk of hospital admissions in older adults with frailty [54].

Bias and confounding pose a significant challenge in observational research studies [55]. Patients with frailty have worse health outcomes compared to those without frailty [56]. It has been suggested that the adverse outcomes of frailty may outweigh the impact of IP. There is a risk of unmeasured confounding in the observational studies which may account for the lack of an association in individual studies or the meta-analysis. Comprehensive adjustment is necessary to determine whether adverse outcomes are related to the medicines or the underlying health state [57]. Future studies may use propensity score analysis to account for the multitude of variables in large complex healthcare datasets [58].

There were no studies that represented middle-aged individuals with frailty. Middle-aged adults have been largely excluded from research relating to IP and frailty. This is despite several frailty parameters being present in more than 20% of middle-aged adults attending an outpatient clinic in one study and being predictive of adverse outcomes [16]. This systematic review only examined hospitalised patients which may have explained the lack of studies examining middle-aged adults who are more likely to receive care in the outpatient setting. Limitations in the definition of frailty in middle-aged adults may also have prevented research in the area. The majority of frailty indices have been developed and validated in older rather than middle-aged adults. It is unclear whether these definitions apply to the middle-aged population [59]. The development and validation of tools to identify frailty in middle-aged adults is required to facilitate research in this area [60]. This is important because the earlier identification of adults developing frailty may provide insights into the aetiological factors and how these can be addressed. Future studies should include a focus on middle-aged adults with frailty including those in the outpatient setting.

There are several limitations of this review. Firstly, there was methodological and clinical heterogeneity between the included studies relating to the design, population, and definition of IP. The lack of a single accepted definition of IP may limit the generalisability of these findings. Secondly, the identified observational studies are at risk of unmeasured confounding that may have affected the results and meta-analysis. Thirdly, combining two risk estimates from the same study carries a risk of bias although sensitivity analysis showed that these did not modify the overall effect size. Finally, there were a limited number of studies amenable to meta-analysis for many of the outcomes. This may have led to limited statistical power and reduces the generalisability of our findings. Furthermore, it is possible that a single study could have had a disproportionate influence on the overall effect size.

## Conclusions

Interventions to reduce IP were associated with reduced mortality compared to usual care in hospitalised older adults with frailty. However, there was considerable heterogeneity between the two interventions studied. The overall effect was driven by one study that included deprescribing in addition to geriatric co-management and an interdisciplinary transitional care intervention. It is unclear whether the reduced mortality was attributable to the medication-related aspect of the intervention. Exposure to Beers criteria medicines was also associated with hospital readmissions in adults with frailty. However, there was no evidence to suggest an association between IP more broadly and adverse outcomes in older adults with frailty. There were no studies assessing the effect of IP on readmissions or mortality in middle-aged adults with frailty.

This literature is limited by varying definitions of IP and no studies that include middle-aged adults. The development of a standardised definition of IP and the validation of frailty indices in middle-aged adults would assist further research. Future studies should assess interventions that reduce IP in isolation to confirm these findings. These studies are needed before interventions to reduce IP are incorporated into routine clinical care if the goal is to prevent readmissions or reduce mortality in older adults with frailty. Further observational studies using validated measures of frailty with comprehensive adjustment for confounders are also needed to confirm these findings and determine which types of IP are associated with adverse outcomes as well as to investigate middle-aged cohorts.

### Supplementary Information

Supplementary Material 1.

Supplementary Material 2.

Supplementary Material 3.

## Data Availability

The datasets generated during the current study are available from the corresponding author on reasonable request.

## Abbreviations

ED: Emergency department
IP: Inappropriate prescribing
JBI: Joanna Briggs Institute
PIM: Potentially inappropriate medicines
PPO: Potential prescribing omissions
PRISMA: Preferred Reporting Items for Systematic Reviews and Meta-Analyses
START: Screening Tool to Alert to Right Treatment
STOPP: Screening Tool of Older Persons' Prescriptions
STOPPFrail: Screening Tool of Older Persons Prescriptions in Frail adults with limited life expectancy
RCT: Randomised controlled trial
