# Supplementary material for: Inappropriate prescribing and association with readmission or mortality in hospitalised older adults with frailty: a systematic review and meta-analysis
Source: BMC Geriatr. 2024 Aug 29;24:718. doi: 10.1186/s12877-024-05297-3 (PMC11363439; doi:10.1186/s12877-024-05297-3)
Supplement: Supplementary file 1 — Supplementary Material 1. [file 12877_2024_5297_MOESM1_ESM.docx]

**Appendix A: Search Strategy**

**MEDLINE (OVID)**

| 1 | exp aged/ or "health services for the aged"/ or "homes for the aged"/ or exp geriatrics/ |
| --- | --- |
| atc | (aged or aging or ageing or elder* or ((old or retired) adj2 (people* or patient* or inpatient* or in-patient* or client* or person* or individual* or wom#n or man or men or age)) or older* or geriatr* or gerontolog* or senior* or senescen* or retiree* or sexagenarian* or septuagenarian* or octagenarian* or nonagenarian* or centenarian* or supercentenarian* or veteran*).tw,kf. |
| 3 | 1 or 2 |
| 4 | middle aged/ |
| 5 | (middle age* adj2 (people* or patient* or inpatient* or in-patient* or client* or person* or individual* or wom#n or man or men or age)).tw,kf. |
| 6 | 4 or 5 |
| 7 | 3 or 6 |
| 8 | exp hospital/ or exp hospital unit/ |
| 9 | (Hospital or hospitali?ed or hospital care or in?hospital or in-hospital or inpatient or admission or ward).tw,kf. |
| 10 | 8 or 9 |
| 11 | Inappropriate prescribing/ or Potentially Inappropriate Medication List/ or exp Drug interaction/ |
| 12 | ((Inappropriate or suboptimal) adj (prescri* or drug* or medicine* or medication*)).tw,kf. |
| 13 | ((Beers* or START* or Screening Tool to Alert to Right Treatment* or STOPP* or Screening Tool of Older Persons* Prescription* or PROMPT* or Prescribing Optimally in Middle-aged People?s Treatment*) adj Criteria).tw,kf. |
| 14 | (Potentially inappropriate medicine* or PIM list* or Medication appropriateness index or PRISCUS* or Potential Prescribing Omission*).tw,kf. |
| 15 | ((Prescrib* or medicine* or medication* or drug*) adj (omission* or conflict* or interaction*)).tw,kf. |
| 16 | (Drug disease interaction* or drug drug interaction*).tw,kf. |
| 17 | (duplication of adj (drug* or medicine* or medication*)).tw,kf. |
| 18 | ((prescrib* or medicine* or medication* or drug*) adj conflict*).tw,kf. |
| 19 | 11 or 12 or 13 or 14 or 15 or 16 or 17 or 18 |
| 20 | exp mortality/ or exp hospitalization/ |
| 21 | (Mortality or Death).tw,kf. |
| 22 | (Hospitali?ation* or admission* or readmission* or re?admission* or emergency department visit* or emergency department revisit* or ED visit* or ED revisit*).tw,kf. |
| 23 | 20 or 21 or 22 |
| 24 | 7 and 10 and 19 and 23 |

**Scopus (Search Article Title, Abstract and Keywords)**

| 1 | (aged OR aging OR ageing OR elder* OR ((old OR retired) W/1 (people* OR patient* OR inpatient* OR "in-patient*" OR client* OR person* OR individual* OR wom*n OR man OR men OR age)) OR older* OR geriatr* OR gerontolog* OR senior* OR senescen* OR retiree* OR sexagenarian* OR septuagenarian* OR octagenarian* OR nonagenarian* OR centenarian* OR supercentenarian* OR veteran*) |
| --- | --- |
| 2 | ("middle age*" W/1 (people* OR patient* OR inpatient* OR in-patient* OR client* OR person* OR individual* OR wom*n OR man OR men OR age)) |
| 3 | 1 OR 2 |
| 4 | (Hospital OR hospitali*ed OR "hospital care" OR "in*hospital" OR "in-hospital" OR inpatient OR admission OR ward) |
| 5 | (Inappropriate W/0 (prescri* OR drug* OR medicine* OR medication*)) |
| 6 | ((Beers* OR START* OR "Screening Tool to Alert to Right Treatment*" OR STOPP* OR" Screening Tool of Older Persons* Prescription*" OR PROMPT* OR "Prescribing Optimally in Middle-aged People*s Treatment*") W/0 Criteria) |
| 7 | ("Potentially inappropriate medicine*" OR "PIM list*" OR "Medication appropriateness index" OR PRISCUS* OR "Potential Prescribing Omission*") |
| 8 | (Prescrib* or medicine* or medication* or drug*) W/0 (omission* or conflict* or interaction*) |
| 9 | ("Drug disease interaction*" OR "drug drug interaction*") |
| 10 | (duplication of W/0 (drug* or medicine* or medication*)) |
| 11 | ((prescrib* or medicine* or medication* or drug*) W/0 conflict*) |
| 12 | 5 OR 6 OR 7 OR 8 OR 9 OR 10 OR 11 |
| 13 | (Mortality OR Death) |
| 14 | (Hospitali*ation* OR admission* OR readmission* OR "re*admission*" OR "emergency department visit*" OR "emergency department revisit*" OR "ED visit*" OR "ED revisit*") |
| 15 | 13 OR 14 |
| 16 | 3 AND 4 AND 12 AND 15 |

**Web of Science (Topic Search)**

| 1 | (aged OR aging OR ageing OR elder* OR ((old OR retired) NEAR/2 (people* OR patient* OR inpatient* OR "in-patient*" OR client* OR person* OR individual* OR wom$n OR man OR men OR age)) OR older* OR geriatr* OR gerontolog* OR senior* OR senescen* OR retiree* OR sexagenarian* OR septuagenarian* OR octagenarian* OR nonagenarian* OR centenarian* OR supercentenarian* OR veteran*) |
| --- | --- |
| 2 | ("middle age*" NEAR/2 (people* OR patient* OR inpatient* OR in-patient* OR client* OR person* OR individual* OR wom$n OR man OR men OR age)) |
| 3 | 1 OR 2 |
| 4 | (Hospital OR hospitali$ed OR "hospital care" OR "in$hospital" OR "in-hospital" OR inpatient OR admission OR ward) |
| 5 | ((Inappropriate or suboptimal)) NEAR/0 (prescri* OR drug* OR medicine* OR medication*)) |
| 6 | ((Beers* OR START* OR "Screening Tool to Alert to Right Treatment*" OR STOPP* OR" Screening Tool of Older Persons* Prescription*" OR PROMPT* OR "Prescribing Optimally in Middle-aged People?s Treatment*") NEAR/0 Criteria) |
| 7 | ("Potentially inappropriate medicine*" OR "PIM list*" OR "Medication appropriateness index" OR PRISCUS* OR "Potential Prescribing Omission*") |
| 8 | ((Prescrib* OR medicine* OR medication* OR drug*) NEAR/0 (omission* OR conflict* OR interaction*)) |
| 9 | ("Drug disease interaction*" OR "drug drug interaction*") |
| 10 | ("duplication of" NEAR/0 (drug* OR medicine* OR medication*)) |
| 11 | ((prescrib* OR medicine* OR medication* OR drug*) NEAR/0 conflict*) |
| 12 | 5 OR 6 OR 7 OR 8 OR 9 OR 10 OR 11 |
| 13 | (Mortality OR Death) |
| 14 | (Hospitali$ation* OR admission* OR readmission* OR "re$admission*" OR "emergency department visit*" OR "emergency department revisit*" OR "ED visit*" OR "ED revisit*") |
| 15 | 13 OR 14 |
| 16 | 3 AND 4 AND 12 AND 15 |

**Cochrane Database – FIXED**

| 1 | [mh ^"aged"] |
| --- | --- |
| 2 | [mh ^"health services for the aged"] |
| 3 | [mh ^"homes for the aged"] |
| 4 | [mh ^"geriatrics] |
| 5 | (aged or aging or ageing or elder* or ((old or retired) NEAR/2 (people* or patient* or inpatient* or in-patient* or client* or person* or individual* or wom#n or man or men or age)) or older* or geriatr* or gerontolog* or senior* or senescen* or retiree* or sexagenarian* or septuagenarian* or octagenarian* or nonagenarian* or centenarian* or supercentenarian* or veteran*):ti,ab,kw |
| 6 | 1 or 2 or 3 or 4 or 5 |
| 7 | [mh ^"middle aged"] |
| 8 | (middle?age* NEAR/2 (people* or patient* or inpatient* or in-patient* or client* or person* or individual* or wom#n or man or men or age)):ti,ab,kw |
| 9 | 7 or 8 |
| 10 | 6 or 9 |
| 11 | [mh ^"hospitals"] |
| 12 | [mh ^"hospital units"] |
| 13 | (Hospital or hospitali?ed or hospital care or in?hospital or in-hospital or inpatient or admission or ward):ti,ab,kw |
| 14 | 11 or 12 or 13 |
| 15 | [mh ^"Inappropriate prescribing"] |
| 16 | [mh ^"Potentially Inappropriate Medication List"] |
| 17 | [mh ^"Drug interaction"] |
| 18 | ((Inappropriate or suboptimal) NEXT (prescri* or drug* or medicine* or medication*)):ti,ab,kw |
| 19 | ((Beers* or START* or Screening Tool to Alert to Right Treatment* or STOPP* or Screening Tool of Older Persons* Prescription* or PROMPT* or Prescribing Optimally in Middle-aged People?s Treatment*) NEXT Criteria):ti,ab,kw |
| 20 | (Potentially inappropriate medicine* or PIM list* or Medication appropriateness index or PRISCUS* or Potential Prescribing Omission*):ti,ab,kw |
| 21 | ((Prescrib* or medicine* or medication* or drug*) NEXT (omission* or conflict* or interaction*)):ti,ab,kw |
| 22 | (Drug disease interaction* or drug drug interaction*):ti,ab,kw |
| 23 | (duplication of NEXT (drug* or medicine* or medication*)):ti,ab,kw |
| 24 | ((prescrib* or medicine* or medication* or drug*) NEXT conflict*):ti,ab,kw |
| 25 | 15 or 16 or 17 or 18 or 19 or 20 or 21 or 22 or 23 or 24 |
| 26 | [mh ^"mortality"] |
| 27 | (Mortality or Death):ti,ab,kw |
| 28 | (Hospitali?ation* or admission* or readmission* or re?admission* or emergency department visit* or emergency department revisit* or ED visit* or ED revisit*):ti,ab,kw |
| 29 | 26 or 27 or 28 |
| 30 | 10 and 14 and 25 and 29 |

**CINAHL (EBSCO)**

| Title and Abstract Search | |
| --- | --- |
| 1 | (MH "Aged+") OR (MH "Health Services for Older Persons") OR (MH "Nursing Homes+") OR (MH "Geriatrics") |
| 2 | TI (aged OR aging OR ageing OR elder* OR ((old OR retired) n2 (people* OR patient* OR inpatient* OR "in-patient*" OR client* OR person* OR individual* OR wom#n OR man OR men OR age)) OR older* OR geriatr* OR gerontolog* OR senior* OR senescen* OR retiree* OR sexagenarian* OR septuagenarian* OR octagenarian* OR nonagenarian* OR centenarian* OR supercentenarian* OR veteran*)  OR  AB (aged OR aging OR ageing OR elder* OR ((old OR retired) n2 (people* OR patient* OR inpatient* OR "in-patient*" OR client* OR person* OR individual* OR wom#n OR man OR men OR age)) OR older* OR geriatr* OR gerontolog* OR senior* OR senescen* OR retiree* OR sexagenarian* OR septuagenarian* OR octagenarian* OR nonagenarian* OR centenarian* OR supercentenarian* OR veteran*) |
| 3 | 1 OR 2 |
| 4 | (MH "Middle Age+") |
| 5 | TI (("middle age*" n2 (people* OR patient* OR inpatient* OR in-patient* OR client* OR person* OR individual* OR wom#n OR man OR men OR age))  OR  AB (("middle age*" n2 (people* OR patient* OR inpatient* OR in-patient* OR client* OR person* OR individual* OR wom#n OR man OR men OR age)) |
| 6 | 4 OR 5 |
| 7 | 3 OR 6 |
| 8 | (MH "Hospitals+") OR (MH "Hospital Units+") |
| 9 | TI (Hospital OR hospitali?ed OR "hospital care" OR "in?hospital" OR "in-hospital" OR inpatient OR admission OR ward)  OR  AB (Hospital OR hospitali?ed OR "hospital care" OR "in?hospital" OR "in-hospital" OR inpatient OR admission OR ward) |
| 10 | 8 OR 9 |
| 11 | (MH "Inappropriate prescribing") OR (MH "Drug interactions+") |
| 12 | TI ((Inappropriate or suboptimal) n0 (prescri* OR drug* OR medicine* OR medication*))  OR  AB ((Inappropriate or suboptimal) n0 (prescri* OR drug* OR medicine* OR medication*)) |
| 13 | TI ((Beers* OR START* OR "Screening Tool to Alert to Right Treatment*" OR STOPP* OR" Screening Tool of Older Persons* Prescription*" OR PROMPT* OR "Prescribing Optimally in Middle-aged People?s Treatment*") n0 Criteria)  OR  AB ((Beers* OR START* OR "Screening Tool to Alert to Right Treatment*" OR STOPP* OR" Screening Tool of Older Persons* Prescription*" OR PROMPT* OR "Prescribing Optimally in Middle-aged People?s Treatment*") n0 Criteria) |
| 14 | TI ("Potentially inappropriate medicine*" OR "PIM list*" OR "Medication appropriateness index" OR PRISCUS* OR "Potential Prescribing Omission*”)  OR  AB ("Potentially inappropriate medicine*" OR "PIM list*" OR "Medication appropriateness index" OR PRISCUS* OR "Potential Prescribing Omission*") |
| 15 | TI ((Prescrib* OR medicine* OR medication* OR drug*) n0 (omission* OR conflict* OR interaction*))  OR  AB ((Prescrib* OR medicine* OR medication* OR drug*) n0 (omission* OR conflict* OR interaction*)) |
| 16 | TI ("Drug disease interaction*" OR "drug drug interaction*")  OR  AB ("Drug disease interaction*" OR "drug drug interaction*") |
| 17 | TI ("duplication of" n0 (drug* OR medicine* OR medication*))  OR  AB ("duplication of" n0 (drug* OR medicine* OR medication*)) |
| 18 | TI ((prescrib* OR medicine* OR medication* OR drug*) n0 conflict*)  OR  AB ((prescrib* OR medicine* OR medication* OR drug*) n0 conflict*) |
| 19 | 11 OR 12 OR 13 OR 14 OR 15 OR 16 OR 17 OR 18 |
| 20 | (MH "Mortality+") OR (MH "Hospitalization+") |
| 21 | TI (Mortality OR Death)  OR  AB (Mortality OR Death) |
| 22 | TI (Hospitali?ation* OR admission* OR readmission* OR "re?admission*" OR "emergency department visit*" OR "emergency department revisit*" OR "ED visit*" OR "ED revisit*")  OR  AB (Hospitali?ation* OR admission* OR readmission* OR "re?admission*" OR "emergency department visit*" OR "emergency department revisit*" OR "ED visit*" OR "ED revisit*") |
| 23 | 20 OR 21 OR 22 |
| 24 | 7 AND 10 AND 19 AND 23 |

**EMBASE (OVID)**

| 1 | exp Aged/ or "frail elderly"/ or exp "elderly care"/ or "home for the aged"/ or exp geriatrics/ |
| --- | --- |
| 2 | (aged or aging or ageing or elder* or ((old or retired) adj2 (people* or patient* or inpatient* or in-patient* or client* or person* or individual* or wom#n or man or men or age)) or older* or geriatr* or gerontolog* or senior* or senescen* or retiree* or sexagenarian* or septuagenarian* or octagenarian* or nonagenarian* or centenarian* or supercentenarian* or veteran*).tw,kf. |
| 3 | 1 or 2 |
| 4 | exp Middle Aged/ |
| 5 | (middle age* adj2 (people* or patient* or inpatient* or in-patient* or client* or person* or individual* or wom#n or man or men or age)).tw,kf. |
| 6 | 4 or 5 |
| 7 | 3 or 6 |
| 8 | exp Hospital/ |
| 9 | (Hospital or hospitali?ed or hospital care or in?hospital or in-hospital or inpatient or admission or ward).tw,kf. |
| 10 | 8 or 9 |
| 11 | exp Inappropriate prescribing/ or Potentially Inappropriate Medication/ or exp Drug interaction/ |
| 12 | ((Inappropriate or suboptimal) adj (prescri* or drug* or medicine* or medication*)).tw,kf. |
| 13 | ((Beers* or START* or Screening Tool to Alert to Right Treatment* or STOPP* or Screening Tool of Older Persons* Prescription* or PROMPT* or Prescribing Optimally in Middle-aged People?s Treatment*) adj Criteria).tw,kf. |
| 14 | (Potentially inappropriate medicine* or PIM list* or Medication appropriateness index or PRISCUS* or Potential Prescribing Omission*).tw,kf. |
| 15 | ((Prescrib* or medicine* or medication* or drug*) adj (omission* or conflict* or interaction*)).tw,kf. |
| 16 | (Drug disease interaction* or drug drug interaction*).tw,kf. |
| 17 | (duplication of adj (drug* or medicine* or medication*)).tw,kf. |
| 18 | ((prescrib* or medicine* or medication* or drug*) adj conflict*).tw,kf. |
| 19 | 11 or 12 or 13 or 14 or 15 or 16 or 17 or 18 |
| 20 | exp Mortality/ or exp Hospitalization/ |
| 21 | (Mortality or Death).tw,kf. |
| 22 | (Hospitali?ation* or admission* or readmission* or re?admission* or emergency department visit* or emergency department revisit* or ED visit* or ED revisit*).tw,kf. |
| 23 | 20 or 21 or 22 |
| 24 | 7 and 10 and 19 and 23 |

**Google Scholar**

((older OR middle aged) AND adult) AND (inappropriate prescribing OR potentially inappropriate medic* OR PIM list* OR prescribing omission OR drug interaction OR duplication of medic*) AND (mortality OR hospitali?ation OR readmission*)

**MEDLINE (OVID) (Update 12 July 2024)**

| 1 | exp aged/ or "health services for the aged"/ or "homes for the aged"/ or exp geriatrics/ |
| --- | --- |
| atc | (aged or aging or ageing or elder* or ((old or retired) adj2 (people* or patient* or inpatient* or in-patient* or client* or person* or individual* or wom#n or man or men or age)) or older* or geriatr* or gerontolog* or senior* or senescen* or retiree* or sexagenarian* or septuagenarian* or octagenarian* or nonagenarian* or centenarian* or supercentenarian* or veteran*).tw,kf. |
| 3 | 1 or 2 |
| 4 | middle aged/ |
| 5 | (middle age* adj2 (people* or patient* or inpatient* or in-patient* or client* or person* or individual* or wom#n or man or men or age)).tw,kf. |
| 6 | 4 or 5 |
| 7 | 3 or 6 |
| 8 | exp hospital/ or exp hospital unit/ |
| 9 | (Hospital or hospitali?ed or hospital care or in?hospital or in-hospital or inpatient or admission or ward).tw,kf. |
| 10 | 8 or 9 |
| 11 | Inappropriate prescribing/ or Potentially Inappropriate Medication List/ or exp Drug interaction/ |
| 12 | ((Inappropriate or suboptimal) adj (prescri* or drug* or medicine* or medication*)).tw,kf. |
| 13 | ((Beers* or START* or Screening Tool to Alert to Right Treatment* or STOPP* or Screening Tool of Older Persons* Prescription* or PROMPT* or Prescribing Optimally in Middle-aged People?s Treatment*) adj Criteria).tw,kf. |
| 14 | (Potentially inappropriate medicine* or PIM list* or Medication appropriateness index or PRISCUS* or Potential Prescribing Omission*).tw,kf. |
| 15 | ((Prescrib* or medicine* or medication* or drug*) adj (omission* or conflict* or interaction*)).tw,kf. |
| 16 | (Drug disease interaction* or drug drug interaction*).tw,kf. |
| 17 | (duplication of adj (drug* or medicine* or medication*)).tw,kf. |
| 18 | ((prescrib* or medicine* or medication* or drug*) adj conflict*).tw,kf. |
| 19 | 11 or 12 or 13 or 14 or 15 or 16 or 17 or 18 |
| 20 | exp mortality/ or exp hospitalization/ |
| 21 | (Mortality or Death).tw,kf. |
| 22 | (Hospitali?ation* or admission* or readmission* or re?admission* or emergency department visit* or emergency department revisit* or ED visit* or ED revisit*).tw,kf. |
| 23 | 20 or 21 or 22 |
| 24 | 7 and 10 and 19 and 23 |
| 25 | limit 24 to dt=20220712-20240712 |
| 26 | 25 and (frail*).tw,kf. |
