# Supplementary material for: Inappropriate prescribing and association with readmission or mortality in hospitalised older adults with frailty: a systematic review and meta-analysis
Source: BMC Geriatr. 2024 Aug 29;24:718. doi: 10.1186/s12877-024-05297-3 (PMC11363439; doi:10.1186/s12877-024-05297-3)
Supplement: Supplementary file 2 — Supplementary Material 2. [file 12877_2024_5297_MOESM2_ESM.docx]

**Appendix B: Data extraction instrument**

| **Study Characteristic** |  |
| --- | --- |
| Year |  |
| First Author |  |
| Country |  |
| Study Design |  |
| Sample Size |  |
| Percentage Male |  |
| Age (mean and SD) |  |
| Population |  |
| Study Duration and/or Follow Up Period |  |
| Intervention (for interventional studies) |  |
| Inappropriate Prescribing Measure or Tool |  |
| Outcomes Measured |  |
| Outcome Results |  |
| Subgroups |  |
| Measure of Frailty |  |
| Statistical Analysis Used |  |
| Adjusted vs Unadjusted Analysis |  |
| Covariates |  |
