# Supplementary material for: Inappropriate prescribing and association with readmission or mortality in hospitalised older adults with frailty: a systematic review and meta-analysis
Source: BMC Geriatr. 2024 Aug 29;24:718. doi: 10.1186/s12877-024-05297-3 (PMC11363439; doi:10.1186/s12877-024-05297-3)
Supplement: Supplementary file 3 — Supplementary Material 3. [file 12877_2024_5297_MOESM3_ESM.docx]

**Supplementary Table 1: Statistical Analysis and Covariates in Included Studies**

| **Study** | **Study Design** | **Adverse Outcomes** | **Statistical Analysis Used** | **Unadjusted or Adjusted Analysis** | **Covariates** |
| --- | --- | --- | --- | --- | --- |
| Bennett et al.  2014 | Prospective cohort | Hospital readmission | Binary logistic regression | Adjusted and unadjusted | Age  Sex  Comorbidities  Living status  ADLs and instrumental ADLs  Investigations of fall  Alcohol use |
| Forget et al.  2020 | Retrospective cohort | ED visits | Multivariate logistic regression | Adjusted | Age  Sex  Charlson comorbidity score  Surgical specialty |
| de Almeida et al.  2020 | Retrospective cohort | Mortality | Time dependent Cox proportional hazard model | Adjusted and unadjusted | Age  Sex  Katz score  eGFR at discharge  CHA2DS2-VASC score |
| Liang et al. 2022 | Retrospective cohort | Readmissions and ED visits | Multivariable logistic regression | Adjusted | Age  Sex  Education level  Marriage status  BMI  Admission route  Charlson comorbidity index  Self-reported visual and hearing impairment  Risk of malnutrition  Risk of pressure sore  Depressive mood  History of fall  Use of physical restraint  ADL scores at discharge  Use of nasogastric tube or Foley's catheter at discharge  Department of admission |
| Cheong et al.  2020 | Case-control | Repeated hospital admissions | Multivariable logistic regression | Adjusted | Age  Sex  Number of comorbidities  Discharged to home  Living in the most deprived areas  Number of PRN drugs  Prescribed diuretics  Hyperpolypharmacy (≥10 medicines) |
